# Autoimmunity Profiles as Prognostic Indicators in Patients with Colorectal Cancer versus Those with Cancer at Other Sites: A Prospective Study

**DOI:** 10.3390/cancers13133239

**Published:** 2021-06-29

**Authors:** Paola Sena, Stefano Mancini, Jessika Bertacchini, Gianluca Carnevale, Monica Pedroni, Luca Roncucci

**Affiliations:** 1 Department of Surgery, Medicine, Dentistry and Morphological Sciences with Interest in Transplant, Oncology and Regenerative Medicine, University of Modena and Reggio Emilia, Via del Pozzo 71, 41124 Modena, Italy; paola.sena@unimore.it (P.S.); jessika.bertacchini@unimore.it (J.B.); gianluca.carnevale@unimore.it (G.C.); 2 Department of Internal Medicine and Rehabilitation, Santa Maria Bianca Hospital, AUSL Modena, Via A. Fogazzaro 6, 41037 Mirandola, Italy; manciobo@gmail.com; 3 Department of Medical and Surgical Sciences, University of Modena and Reggio Emilia, Via del Pozzo 71, 41124 Modena, Italy; monica.pedroni@unimore.it

**Keywords:** autoimmunity, autoantibodies, cancer, prognosis, prospective study

## Abstract

**Simple Summary:**

The clinical utility of tumor-associated autoantibodies (TAABs) detected in patient sera with different types of cancer has not yet been established. Their possible use in early cancer detection, oncological follow-up, and patient prognosis is highly desirable. We developed a prospective study to investigate the role of TAABs in a five-year survival analysis in different types of cancer patients. Overall, overproduction of TAABs is associated with advanced oncological disease, the presence of metastasis, and poorer prognosis of cancer patients. There is evidence that more intensive follow-up programs provide different results for colorectal cancer than other cancers, because more intensive follow-up improves survival and is cost-effective in colorectal cancer. It is necessary to emphasize that there are many important aspects of follow-up in addition to detection of recurrence, and this must lead to proposals to change the way follow-up care is delivered.

**Abstract:**

Colorectal cancer represents a paradigmatic model of inflammatory carcinogenesis accompanied by the production of several kinds of tumor-associated autoantibodies (TAABs). The specific aim of this study is to define the clinical impact of the presence of non-specific circulating TAABs in a cohort of cancer patients and to establish whether significant differences were present between colorectal cancer and cancers at other sites. For this aim a prospective study was developed and a five-year survival analysis performed. Indirect immunofluorescence on rat tissues for non-organ specific autoantibodies (NOSAs: liver-kidney-stomach), on rat colon substrates (colon-related autoantibodies, CAAs) and on HEp-2 cell lines was performed. NOSA positivity was more frequent in patients with colorectal cancer than in those with cancer at other sites. Survival analysis demonstrated a significantly worse prognosis in cancer patients positive for TAABs. CAA positivity is a predictor of survival, independently from the presence of comorbidities, and HEp-2 reactivity was a strong predictor of survival in a stepwise Cox-regression model, including stage at diagnosis. Overall overproduction of TAABs is associated with advanced oncological disease, the presence of metastasis, and poorer prognosis of cancer patients.

## 1. Introduction

The worldwide cancer burden in 2020, based on GLOBOCAN cancer incidence and mortality estimates produced by the International Agency for Research on Cancer, shows that there were approximately 19.3 million new cases and 10 million deaths from cancer around the world in 2020. Lung cancer remained the leading cause of cancer death, with an estimated 1.8 million deaths (18%), followed by colorectal (9.4%), liver (8.3%), stomach (7.7%), and female breast (6.9%) cancers [1].

Up to half of all cancers are preventable [2], so primary prevention including the discovery of effective biomarkers is a key factor in cancer control.

It is well-established that neoplasms induce the release of proteins, which triggers an immune response in humans. During this response, the immune system performs very efficient biological amplification, using antigenic tumor proteins as templates. Very small amounts of antigen can be indirectly detected [3,4,5,6]. It is interesting to note that a considerable number of autoantibodies is shared by autoimmune diseases and cancer. In fact, about 30% of all cancer patients have anti-nuclear antibodies (ANA) circulating in the serum [7]. Among these, those affected by colorectal cancer have been well characterized and studied [8,9]. These autoantibodies are similar to those associated with Sjögren’s syndrome, and systemic lupus erythematosus, while they are generally absent or present in very low concentrations in healthy individuals [10].

The induction of inflammation in the tumor microenvironment could be the cause of the intracellular antigens release with consequent abnormal production of autoantibodies in cancer patients [11]. In these patients, the formation of autoantibodies can be explained in different ways, such as being triggered by new or altered antigens produced by the tumor itself (tumor associated antigens, TAA), or by antigens generally hidden from our immune system [6]. The clinical utility of tumor-associated autoantibodies (TAABs), detected in patient sera with different types of cancer has not yet been established. Their possible use in early cancer detection [12,13], oncological follow-up, and patient prognosis [14,15] is highly desirable. Systematic studies addressing the presence of non-specific autoimmune hallmarks in cancer patients are lacking. Instead, they could be interesting because TAABs can be easily extracted from the serum by minimally invasive blood sampling. Moreover, it has been demonstrated that their levels increase from the earliest stages of tumorigenesis [16] and are observed in patients with different carcinomas, including gastrointestinal [16], lung [17], breast [18], ovarian [19], and prostate [20]. Most notably, their presence in the blood may be revealed several months or years before clinical confirmation of a tumor [21].

Autoimmune response, such as autoantibody production, may be part of a chronic inflammatory response to cancer cells. The induction of inflammation in the tumor microenvironment facilitates release of intracellular antigens resulting in abnormal exposure to autologous antigens to the immune system, which may provide an explanation for the large number of autoantibodies produced in cancer patients [22].

Colorectal cancer represents a paradigmatic model of inflammatory carcinogenesis accompanied by the production of several kinds of TAAs and TAABs [23]. Inflammatory changes in the colonic mucosa trigger and promote gradual dysplastic alterations in the colonic mucosa [24,25]. The development of a humoral immunological response may reflect the activation of the immune system against the expression of altered antigens during the adenoma-carcinoma sequence. Indeed, recent works have highlighted the possible usefulness of serum biomarkers related to autoimmunity in colorectal cancer screening and even in normal subjects [26,27].

This peculiar characteristic of colorectal cancer has driven our attention to consider possible different steps leading to humoral responses in CRC patients compared with patients affected by cancer at other sites. Thus, we set out to design a study with two main aims: (1) to define the clinical impact of the presence of non-specific circulating TAABs in a cohort of cancer patients, and (2) to establish whether significant differences were present between CRC and other cancer patients. For this purpose, a prospective study was designed to investigate the role of non-specific TAABs in a five-year survival analysis.

## 2. Materials and Methods

### 2.1. Design of the Study and Ethics Statement

We aimed at investigating the prognostic value of non-specific TAABs in a prospective study of survival (five-year follow-up). The study was approved by a competent Ethics Committee, and the Local Health Agency of Modena. Every patient enrolled in the study signed a detailed written informed consent. The study was carried out according to the Declaration of Helsinki, the Good Clinical Practice principles for medical research, and the current regulations relating to the protection and processing of personal and sensitive data (European Regulation n. 679/2016).

### 2.2. Patients

A cohort of 128 patients (81 males and 47 females) were enrolled from a pool of patients consecutively referred to the University Hospital of Modena for a period ranging from June 2009 to January 2011 and characterized by a history or new evidence of neoplastic disease. Criteria of inclusion or exclusion are the following: for inclusion, age > 17 years, histology positive for cancer, informed consent; for exclusion, sepsis, severe prognosis (expected death within 48 h), severe renal impairment (Glomerular Filtration Rate, GFR <11 mL/min), dialysis, Child–Pugh score >9, blood/blood products infusion in the last 2 weeks.

A blood draw was taken once only at the time of enrollment. Blood samples were immediately processed for serum extraction by centrifugation, and stored frozen at −80 °C until test execution. Patients were fasting from 8 h. For each patient, a detailed medical history was obtained with a particular focus on the oncological disease (cancer type with documented histology, stage of disease according to TNM, age of onset, previous chemo- or radio-therapy), including a scale for the assessment of patient comorbidities (Cumulative Illness Rating Scale, CIRS) [28]. All clinical data were recorded anonymously.

### 2.3. Substrates and Tests

Indirect immunofluorescence (IFA) on rat tissues for non-organ specific autoantibodies (NOSAs: liver-kidney-stomach), on rat colon substrates (colon-related autoantibodies, CAAs), and on HEp-2 cell lines was performed.

Non-organ-specific substrates were prepared according to standard recommendations in liver autoimmune serology, as previously reported [29], using rat liver-kidney-stomach (LKS) substrates. Substrates were taken from 12-week-old female rats weighing 250/300 g (Charles River laboratories, Calco, Lecco, Italy) used for a parallel study [30]. Animal care, maintenance, and surgery were conducted in accordance with Italian law (D.L. number 116/1992) and European legislation (EEC number 86/609). All experimental protocols were reviewed by the Local Animal Ethics Committee (OPBA), the University of Modena and Reggio Emilia, Italy, and approved by Ministero della Salute (authorization number 556/2017-PR released on 7 June 2017).

LKS substrates taken from 9 rats were immediately fixed in 4% paraformaldehyde for 1 h and embedded in paraffin wax. Routine histology of all tissue samples was carried out with haematoxylin and eosin (H&E) staining. For immunofluorescence analysis, 3 µm sections were placed on SuperFrost Plus microscope slides (Menzel-Gläser).

Colon tissue substrates were used from commercial slides (Rat Ileum Colon Slides, Ref. ICR4V, Astra S.r.l., Milan, Italy). Antinuclear antibodies (ANAs) and cytoplasmic reactivity were tested on HEp-2 cell lines commercially available for ANA testing (Kallestad HEp-2 Slides #26104, Bio-Rad, Milan, Italy).

Immunoblot tests were also performed on specific positive sera for some characteristic patterns of reactivity (e.g., anti-mitochondrial antibodies and anti-centromere antibodies). We used the following commercial kits: Liver Profile 2, Ref. DL 1300-1601-2G, Euroimmun Italia S.r.l, and ANA Profile 3, Ref. DL 1590-1601-3 G, Euroimmun Italia S.r.l.

### 2.4. Dilution of Sera and Fluorochrome-Labelled Reagents

Conventionally, a serum dilution of 1:10 is frequently associated with positive reactivity, even in sera from healthy adults, so that a clinically significant level of positivity would start at the arbitrary dilution of 1:10. Thus, we used a 1/40 dilution of the sera in phosphate buffered saline (PBS). Fluorescein-conjugated anti-human immunoglobulin diluted 1:100 (Anti-Human Polyvalent Immunoglobulin IgA, IgG, IgM FITC Conjugate, Sigma ImmunoChemicals, St. Louis, MO, USA) was used as a secondary antibody. A solution containing 30 μL of test and control sera diluted in PBS pH 7.2 was applied to the slide to cover the entire tissue section. The sections were left for 30 min at room temperature in a humidified box, and then washed in an excess of PBS with mild shaking. The sections were then exposed to fluorochrome-labelled anti-human IgG, IgA, and IgM antiserum, or fluorochrome-labelled antiserum to human IgG only, for an additional 30 min at RT in the humidified box and then re-washed as above. The slides were then mounted with anti-fading medium (0.21 M DABCO and 90% glycerol in 0.02 M Tris, pH 8.0).

The immunofluorescence patterns were assessed under a fluorescence microscope (Orthoplan, Leitz, Wetzlar, Germany) and confocal imaging was performed on a Leica TCS SP2 AOBS confocal laser scanning microscope. The confocal serial sections were processed with the Leica LCS software to obtain three-dimensional projections. Image rendering was performed by adobe Photoshop software.

A code number was assigned to each condition and slides were analyzed in a blinded fashion by two different operators experienced in immunofluorescence microscopy. In case of discrepancy (10% of all), a third blinded experienced operator was consulted separately from the first two.

### 2.5. Types of Reactivity

Non-Organ Specific Autoantibodies (NOSAs) were evaluated on LKS substrates according to the classic patterns recognized in liver autoimmune diseases [29] and in the literature [31]. We did not aim to recognize specific antigens or autoantibodies; thus, if a pattern was not clearly identified, we also used a descriptive nomenclature. This was particularly true for non-specific staining of the hepatocyte cytosol that did not match specific patterns of reactivity on the liver-kidney-stomach substrates (as happens, for example, with the liver cytosol-1 antibody, LC-1, or liver-kidney microsomal antibodies, LKM-1/2, etc.).

HEp-2 cells were evaluated both for nuclear and cytoplasmic reactivity, according to the experience of the authors and the commonly used nomenclature [32,33].

Since a specific nomenclature of colon-related autoantibodies does not exist, we defined a descriptive method of the main patterns of reactivity as follows: (1) D-ANA for diffuse antinuclear staining in the rat colon tissue, both in the stroma and in the muscles and in the nuclei of epithelial cells; (2) MS-ANA: ANA with a much more relevant intensity in the nuclei of muscle and stromal/interstitial cells; (3) GE-ANA: ANA staining with a predilection of glandular epithelial antinuclear staining; (4) SMA: staining of the smooth muscle fibers; (5) SITA: staining of the stromal and interstitial fibers; (6) CMPA: staining of the myenteric colon plexus; (7) CECA: staining localized at the colon epithelial cell cytoplasm.

### 2.6. Survival Analysis

Enrolled patients were followed-up with until 31 May 2016. Survival status was extracted from the clinical record archive of the University Hospital of Modena.

### 2.7. Statistical Analysis

Differences among groups were tested through *t*-tests or Mann–Whitney rank sum tests according to data distribution. Z-tests and Chi-square tests were performed to compare proportions in the number of observations for each category and dichotomous variables. Regression analyses were used to measure the strength of association between pairs of variables. The odds ratio for an estimate of the odds of an event occurring in the different groups was calculated for positive and negative patients in the autoantibody testing. For analyzing the occurrence of death during the five-year follow-up, a survival Log-Rank test and Kaplan–Meier curves were developed. In order to define the relative weight and the best predictors among the variables implicated in patients’ survival, a Cox-regression proportional hazards model was created. Statistical analyses were performed through the statistical software platform SigmaPlot© v.12, Systat Software, Inc. *p* < 0.05 was considered significant.

## 3. Results

### 3.1. Clinical Characteristics of Patients

Clinical characteristics of the cohort of patients selected are summarized in Table 1. Mean age at enrollment was 73.5 years, with a higher prevalence of male (63.3%) and advanced stage (III and IV) tumors (61.7%). In total, 75% of the patients had active disease at the time of enrollment, and 28.2% had at least one previous chemotherapy/radiotherapy treatment.

Dividing the cohort into the two main groups (colorectal cancer group (CRCG) and other cancer group (OCG)), there were differences between the two groups regarding the stage of disease (significantly higher prevalence of stage IV patients with higher mortality in OCG with respect to CRCG) and general health status (higher CIRS in OCG than CRCG).

### 3.2. Features and Quantification of Non-Organ Specific Autoantibody (NOSA) Expression Pattern

Considering all types of positive staining, we reported a high occurrence (about 70%) of patients positive for NOSAs in our cohort of patients, as shown in Table 2.

**Figure 1:**
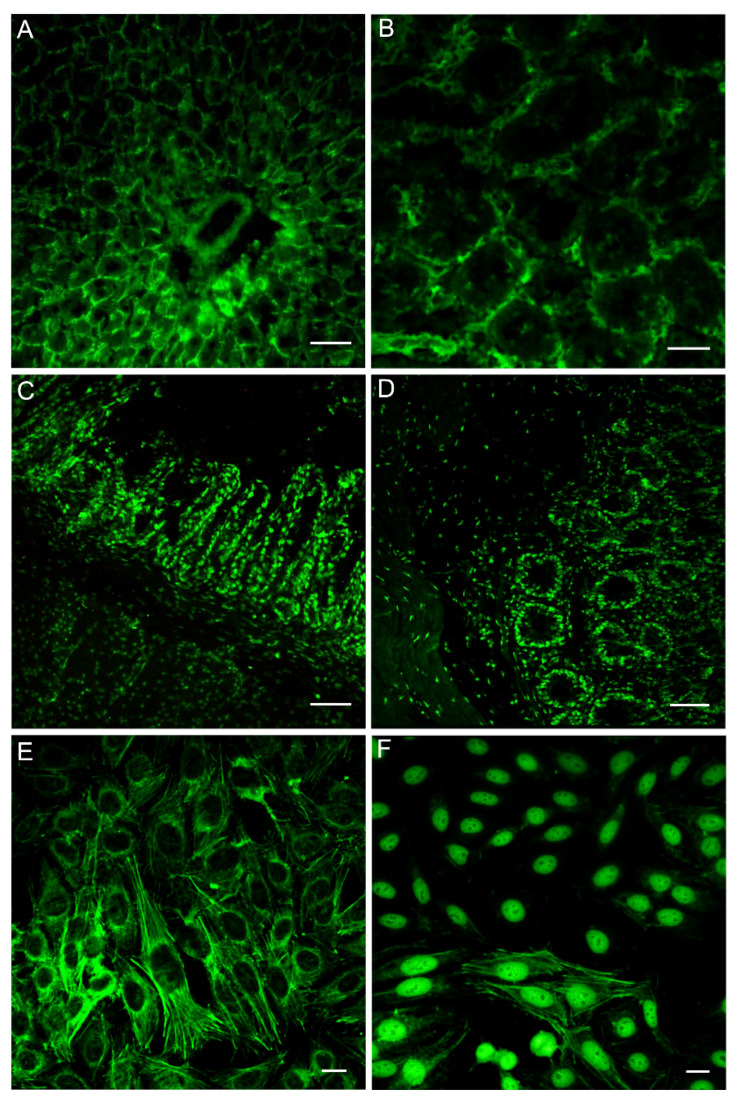
NOSAs, D-ANA and HEp-2 cells immunofluorescence staining patterns of patient with colorectal cancer using an indirect immunofluorescence method. (**A**) Staining surrounding the liver parenchyma, sinusoid, and portal vein was evident using the rat liver substrate. (**B**) Staining of peritubular areas and brush border reactivity was observed using the rat kidney substrate. (**C**) Staining of epithelial cells at nuclear level in the gastric glands and of smooth muscle nuclei was observed using the rat stomach substrate. (**D**) Nuclear staining of epithelial cells into the crypts was very evident as well as the nuclear positivity of the two layers of smooth muscle. (**E**) Representative images of major HEp-2 cell patterns like cytoplasmic fibrillar linear were observed. (**F**) Nuclear dense fine speckled and cytoplasmic fibrillar linear patterns coexisted using the same patient’s serum. Scale bar: (**A**,**B**) = 30 µm; (**C**,**D**) = 50 µm; (**E**,**F**) = 10 µm.

Antinuclear antibodies and smooth muscle antibodies were the most represented, but also a significant number of non-specific staining was found, such as anti-reticulin R2/RS patterns and renal brush border reactivity (Figure 1A–D). The same patient could have more than one positivity. Thus, the sum of the percentages was more than one hundred percent.

Positive NOSAs resulted significantly higher in the OCG than in the CRCG (81.5% vs. 58.7%, respectively, *p* = 0.009). No significant difference of a specific pattern was found between the two groups.

Since it was evident that CRCG differed from OCG in NOSA positivity, we analyzed several variables in the two groups according to NOSA positivity, as shown in Table 3. Specifically, there were differences between patients with positive NOSAs among the two groups for mean CIRS Score (12.8 ± 3.8 and 17.3 ± 4.4, CRCG and OCG, respectively, *p* < 0.001) and CIRS Index, as well as stage III (*p* < 0.001, more patients with positive NOSAs in the CRCG) and stage IV (*p* = 0.01, more patients with positive NOSAs in the OCG) exitus (*p* = 0.006), but not when comparing early vs. advanced stages, and stage I or II alone.

**Figure 2:**
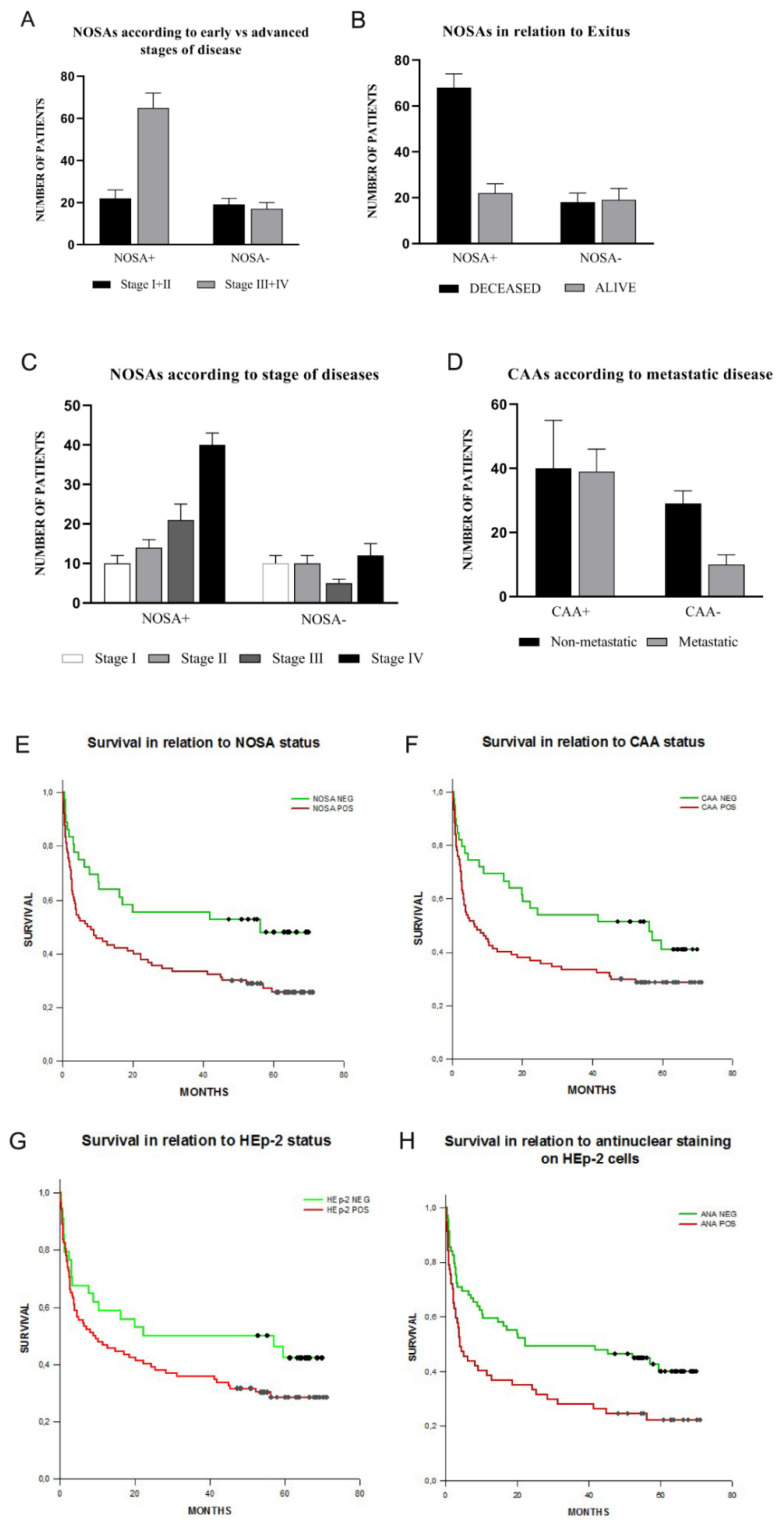
(**A**) The NOSA^+^ (non-organ specific autoantibodies) condition significantly increases the odds of being in advanced stages by 2.71-fold (95% CI: 1.23–5.97) in the cohort of patients (*p* = 0.021). (**B**) NOSA^+^ status increases the odds of the exitus event by 2.75-fold (95% CI: 1.25–6.05, *p* = 0.019) (**C**). NOSA^+^ status significantly increases in number from stage I to stage IV in the entire cohort of patients (Regression analysis, *p* < 0.05). (**D**) The CAA^+^ (colon-related autoantibodies) status significantly increases the odds to have a metastatic disease by a factor of 2.49 (95% CI: 1.10–5.63, *p* = 0.043) among our study population. (**E**) Kaplan-Meier survival analysis for the five-year follow-up demonstarted better prognosis for NOSA- (non-organ specific autoantibodies) negative (Log-Rank test *p* = 0.015) and (**F**) CAA- (colon-specific autoantibodies) negative (Log-Rank test *p* = 0.038) pa-tients. (**G**) HEp-2 status did not reach the statistical significance for differences in survival (Log-Rank test *p* = 0.125). (**H**) The Log-Rank statistics for the survival curves confirmed a significant difference (*p* = 0.009) in survival considering only antinuclear reactivity (dif-fuse or speckled) on HEp-2 cells.

It is noteworthy that, when summarizing the results according to dichotomous variables in the entire cohort of 128 patients in relation to the presence of NOSAs, the advanced stages of disease (stages III and IV) and death were significantly associated to a NOSA-positive status. In particular, a NOSA-positive condition significantly increased the odds of being in advanced stages by 2.71-fold (95% CI: 1.23–5.97, *p* = 0.021), as illustrated in Figure 2A, and increased the odds of the exitus event by a factor of 2.75 (95% CI: 1.25–6.05, *p* = 0.019), as illustrated in Figure 2B.

Regression analyses demonstrated a lack of association between NOSA positivity and patients’ age (*p* = 0.82), as well as between NOSA positivity and CIRS Score (*p* = 0.20); whereas it confirmed the significant association between NOSA positivity and stage of cancer disease (*p* < 0.05), as reported in Figure 2C.

Interestingly, NOSA positivity, when adjusted for tumor stage, showed that a significant difference between CRCG and OCG was evident in stage II and III, suggesting that NOSA positivity is a negative prognostic factor.

### 3.3. Correlation between HEp-2 Cell Substrates Positivity and Patient Clinical Data

Positivity on HEp-2 cell substrates was widely represented (74% of the 128 patients), and the most representative patterns of expression were the following: nuclear dense fine speckled, cytoplasmic fibrillar linear, and non-specific cytoplasm reactivity (Figure 1E,F). Several patterns may coexist in the same patient. Overall, HEp-2 positivity showed no difference between CRCG and OCG. Furthermore, analyzing for particular staining, we found that non-specific cytoplasm positive patterns were significantly more represented in the OCG than in the CRCG (*p* < 0.04) and, in particular, homogenous staining of the cytoplasm occurred in the OCG only (*p* < 0.03).

No dichotomous variable showed significant differences between HEp-2 positive and negative patients, considering the entire cohort of 128 subjects. The analysis of subgroups of positive HEp-2 patients revealed that positive subjects in the CRCG were characterized by a lower CIRS score than that in patients belonging to OCG (12.9 ± 4.4 and 16.9 ± 4.8, CRCG and OCG respectively, *p* < 0.001). Moreover, an increased prevalence of positivity on HEp-2 cells in the metastatic CRCG patients was noted (94.1% and 63.9%, CRCG and OCG, respectively, *p* = 0.02). The presence of an active disease was also associated with a significantly greater number of positive staining in the CRCG than in the OCG (85.4% and 68.7%, CRCG and OCG, respectively, *p* < 0.05).

Regression analyses demonstrated a lack of association between overall HEp-2 positivity (nuclear plus cytoplasmic) and patients’ age (*p* = 0.19), sex (*p* = 0.86), stage of cancer disease (*p* = 0.95), CIRS score (*p* = 0.21), and activity of disease (*p* = 0.23). However, if only nuclear positivity (either diffuse or speckled) is considered, it is noteworthy that a significant direct association was found with the patients’ age (*p* < 0.009).

### 3.4. Colon-Associated Autoantibodies (CAAs) Trend

Unlike the other assays, we found that anti-nuclear staining of this substrate could vary in intensity according to the different types of cells. In particular, we reported that some sera tended to react towards specific nuclei and not (or with a markedly lower intensity) towards others. Specifically, we identified different reactivity in intensity allowing us to define (1) glandular epithelial cell nuclear staining (GE-ANA), (2) muscle/stromal cell nuclear staining (MS-ANA), (3) diffuse nuclear staining (D-ANA) (Figure 1).

CAA positivity was found in 68.7% of the subjects, with no substantial difference between CRCG and OCG. The prevalent types of reactivity were given by antinuclear antibodies (32.8%), staining of the stromal and interstitial fibers (28.9%), and anti-smooth muscle antibodies (12.5%). An interesting difference between CRCG and OCG was observed with regard to the glandular epithelial ANA staining, which was significantly more represented in the CRCG than in OCG (12.7% and 3.1%, respectively, *p* < 0.05). Subgroup analysis of only positive CAA tumors showed higher mean values of the CIRS score in OCG than in CRCG. The OCG group had a significantly lower CIRS score and CIRS index.

χ^2^-square test for dichotomous variables demonstrated a statistically significant difference in CAA positivity between metastatic and non-metastatic patients. The odds ratio calculation showed a 2.49-fold increase (95% CI: 1.10–5.63, *p* = 0.043) for having a metastatic disease in CAA-positive patients among our population, as illustrated in Figure 2D.

Regression analyses demonstrated a lack of association between overall CAA positivity and sex (*p* = 0.27), stage of disease (*p* = 0.08), patients’ age (*p* = 0.18), CIRS score (*p* = 0.93), and the activity of disease (*p* = 0.11), whereas an interesting association was found between CAA positivity and patients’ age (*p* = 0.021).

### 3.5. Survival Outcome Analysis

The Log-Rank statistics for the survival curves demonstrated that there was a statistically significant difference between NOSA-positive and NOSA-negative patients for survival (Figure 2E, *p* = 0.015). Statistical significance was also found analyzing survival curves for CAA-positive and negative patients (Figure 2F, *p* = 0.038), whereas it was not confirmed on HEp-2 substrates (Figure 2G, *p* = 0.125). Interestingly, if we consider only antinuclear staining (either diffuse or spotted) on HEp-2 substrates, the prognostic trend turns out to be strongly statistically significant (*p* = 0.007), with a poorer outcome for ANA positive patients, as shown in Figure 2H.

Using a Cox-regression proportional hazards model with a stepwise method including NOSA, HEp-2, and CAA positivity, patients’ age, stage of disease and CIRS score, the covariate that mostly contributed to patient survival amongst our autoantibody testing was negative HEp-2 test (HR = 0.396, 95% CI: 0.224–0.699, *p* = 0.001), as summarized in Table 4.

## 4. Discussion

In recent years, much attention has been given to the research involved in identifying biomarkers useful in cancer prevention. Part of the focus has been on screening patients who may develop the disease by monitoring their autoantibody profiles prior to the clinical manifestation of symptoms. It has already been shown that autoantibodies can be detected in high-risk patients who have no clinically detectable cancer [34,35]. Therefore, autoantibody signature represents a potentially applicable non-invasive test for screening the high-risk population, as a complement to other screening tests such as colonoscopies for large bowel cancer. In this context, we have developed a prospective study of non-specific autoantibody responses in patients affected by different types of cancer and an evaluation of its possible efficacy in the clinical setting through a five-year survival analysis. Cancer tissues trigger humoral immune responses towards tumor-associated antigens as well as towards self-antigens, producing tumor-associated autoantibodies (TAABs). TAABs were demonstrated to affect (1) early tumor cell clearance (immune surveillance) [36], (2) clonal selection of tumor cells [37,38], (3) cancer promotion through inflammation or post-transcriptional modulation (e.g., anti-p53) [5,39], (4) cancer development through increased oxidative stress in the tumor environment [15]. Complement can facilitate the silent removal of cancer cells and have a dual role in promoting and inhibiting tumor growth; whilst immune response-generating autoantibodies that recognize opsonized fragments could induce a pro-inflammatory and cytotoxic tumor microenvironment [40]. They can also enhance antigen cross-presentation and activation of T lymphocytes or directly bind to cell surface receptors or growth factors, thus interfering in receptor/ligand interactions triggering humoral immune response [5]. These findings have led to thinking of a general protective, rather than an adverse, role of TAABs, despite the conflicting evidence.

This study clearly demonstrates that the presence of non-specific circulating TAABs has a negative role for cancer patients, with severe implications for prognosis. Our data show that patients with NOSA and CAA reactivity are characterized by an advanced stage of disease, poorer prognosis, and the presence of metastasis. The importance of these findings resides in the fact that we considered the presence of TAABs in the widest range as possible. The need to know when autoantibodies appear during tumor progression and what are the pathological stimuli that trigger this response are of fundamental importance when considering them as diagnostic or prognostic biomarkers. To address these issues, the presence of non-specific circulating TAABs and types of reactivity were correlated with the clinical and pathological characteristics of the patients divided into two groups —CRCG, colorectal cancer group, and OCG, other cancer group—since the patients were enrolled at various times and not necessarily at the time of cancer diagnosis. To date, it is still controversial as to whether an increase or decrease in TAABs is beneficial for overall patient survival and appears to differ for each TAAB. For example, increases in autoantibody (AAB) levels may be associated with complete tumor remission in some anticancer therapies [41]. Whereas, in some cases, decreases in AAB levels were observed with cancer progression [42]. Quantification of TAAB levels can be a valuable aid in monitoring the immune response when evaluating the efficacy of existing and new therapeutic agents, and may prove effective as a predictor of recurrences and a favorable clinical outcome. Thus, we did not focus on specific sets of TAABs, but, on the contrary, we sought to define whether a cancer patient who develops a higher autoimmune response is in a better or worse condition than someone with a low response. Here, the evidence is that a more severe disease is linked to a broader activation of the humoral immune response. Indeed, Kaplan–Meier survival curves demonstrate how NOSAs, CAA, and HEp-2 (for the latter antinuclear positivity only) are associated with a decreased survival during the five-year follow-up period.

As mentioned above, current literature reports conflicting data on the prognostic role of specific TAABs in several types of cancer [43]. Anti-p53 antibodies are among the most studied, and a large systematic review clearly highlighted the correlation between p53-specific autologous antibodies and decreased overall, and progression-free, survival in breast, lung, colon, and oral cancer [44]. Interestingly, it has been shown that anti-p53 antibodies can stain different structures, such as centrosomes and mitotic spindle apparatus, in different cell lines [45], though with lower intensity and in fewer steps of the cell cycle than anti-β-tubulin antibodies do. In our study, we have not evaluated anti-p53 immunoreactivity, however we found many different kinds of staining, and two of them were significantly different between the colorectal cancer group (CRCG) and other cancer group (OCG). In fact, CRCG showed a higher prevalence of antinuclear reactivity in the glandular epithelium of the rat colon substrates, while OCG showed a significantly higher prevalence of non-specific cytoplasmic staining on HEp-2 cells. A suggestive hypothesis is that, as it happens for anti-p53 antibodies, different substrates have very different abilities to unmask the great number of circulating TAABs, and the same TAAB at the same titer may furnish not only a significantly different intensity but also a different kind of reactivity according to substrates. Thus, analyzing the impact of TAABs on survival, the Cox-regression model and the multivariate linear regression analysis showed that the best predictors for a *quoad vitam* prognosis are given by a negative result at HEp-2 cell assay (HR 0.396, *p* = 0.001), along with a low stage of disease and low scores on the comorbidity index (CIRS); while CAA status represents the only independent predictor of survival (*p* = 0.035), being that CAA positivity is the only autoantibody test able to discriminate between metastatic and non-metastatic patients. These properties belonging to CAA status may depend on the unique condition that characterizes colon physiology, as the colon is naturally interposed between a rich bacterial flora in the lumen and sterility in the inner vascular and stromal side [46,47]. In some autoimmune diseases of the gastrointestinal tract, such as autoimmune atrophic gastritis (AAG), autoimmune enteropathy (AIE), and ulcerative colitis (UC), the underlying inflammatory process is supported by increased mucosal activation and infiltration of T helper cells, type 1 (Th) 1/Th17 [48]. In these conditions, specific circulating autoantibodies are present, and although their pathogenetic role is still to be clarified, it is well known that immune mechanisms are involved in the progression of chronic inflammation to cancer, i.e., colorectal carcinoma in UC [49]. A recent paper [50] correlates a particular posttranslational modification, arginine deimination, with biomolecular mechanisms involved in the generation of autoantibodies and exacerbation of the inflammatory response, which are involved in the progression of the tumor process. In addition to its role in autoimmunity, evidence from multiple translational and clinical studies indicate the involvement of arginine deimination in inflammatory bowel disease [51]. Most interestingly, a downregulation of intertumoral PAD2 enzymes, involved in the arginine deimination process, has been identified, and is correlated with a worse prognosis in a cohort of colorectal cancer patients compared to the normal mucosa of healthy control tissues [52]. Among the substrates, colon tissue may represent the only one able to unmask TAABs implicated in the epithelial-mesenchymal transition processes, which seem to be a fundamental step in the progression and metastasizing ability of tumors [53].

## 5. Conclusions

Even though this work provides well-defined evidence of the general prognostic role of the presence of increased humoral immune response in cancer patients, the main drawback of the study is that a single blood draw was taken at the enrollment and no further immunochemical follow-up was available; thus, it is impossible to determine whether a patient who resulted positive then turned out negative, or vice versa. This could be interesting for a better definition of survival among positive patients, possibly answering the question about positive patients who survived (and, on the other hand, negative patients who died).

Despite this, our study strongly suggests that the overproduction of nonspecific TAABs is an adverse event in cancer patients and, if confirmed by wider population-based studies, this finding opens up the intriguing possibility of using non-specific circulating TAABs as an effective, reliable, non-invasive, and low-cost marker of prognosis for all types of cancer patients.

## Figures and Tables

**Table 1:** Demographic and clinical characteristics of patients. Legend: CIRS, Cumulative Illness Rating Scale; CRCG, colorectal cancer group; M, mean; n, number of patients; OCG, other cancer group; SD, standard deviation. * For age of patients and stage of disease, χ^2^-tests for trend were used.

Variables	All Patients (N = 128)	CRCG (N = 63)	OCG (N = 65)	*p*
Age, M (±SD)	73.5(±13.5)	73.5(±13.9)	73.5(13.1)	0.99
Male, n (%)	81(63.3)	37(58.7)	44(67.7)	0.38
Age in years, n (%)				
30–45	4(3.1)	2(3.2)	2(3.1)	1.00 *
46–65	29(22.6)	14(22.2)	15(23.1)	
66–85	74(57.8)	37(58.7)	37(56.9)	
>85	21(16.4)	10(15.9)	11(16.9)	
Stage, n (%)				
1	21(16.4)	14(22.2)	7(10.8)	<0.001 *
2	24(18.7)	11(17.5)	13(20.0)	
3	26(20.3)	21(33.3)	5(7.69)	
4	53(41.4)	17(27.0)	36(55.4)	
Not available	4(3.1)	0(0.0)	4(6.1)	0.13
Non-metastatic [I + II + III], (%)	71(55.5)	46(73.0)	25(38.5)	<0.001
Onset time, M in years (±SD)	1.7(±2.8)	1.7(±3.1)	1.7(±2.5)	0.97
Active disease, n (%)	96(75.0)	48(76.2)	48(73.8)	0.75
Deceased, n (%)	85(66.4)	34(54.0)	51(78.5)	0.006
Mean survival, months (±SD)	27.2(±27.1)	37.1(±26.8)	17.9(±24.1)	<0.001
Previous CT/RT, n (%)	36(28.2)	16(25.4)	20(30.8)	0.49
CIRS Score, M (±SD)	15.1 ± 4.9	13.0 ± 4.3	17.2 ± 4.5	<0.001
CIRS Index, M (±SD)	1.08 ± 0.3	0.93 ± 0.3	1.23 ± 0.3	<0.001

**Table 2:** Trends in non-organ specific autoantibodies (NOSAs) according to types of reactivity and the two groups of patients (CRCG, colorectal cancer group, and OCG, other cancer group). Abbreviations: AMA, antimitochondrial antibodies; ANA, antinuclear antibodies; APCA, anti-gastric parietal cell antibodies; GMPA, gastric myenteric plexus antibodies; LCS, liver cytosol staining; SMAV, vascular smooth muscle antibodies; SMAT, tubular smooth muscle antibodies; R2/RS, antireticulin R2/RS pattern; RBB, renal brush border staining.

Variables	All Patients (N = 128)	CRCG (N = 63)	OCG (N = 65)	*p*
NOSA-Pos, n (%)	90(70.3)	37(58.7)	53(81.5)	0.009
ANA	32(25.0)	13(20.6)	19(29.2)	0.26
SMAV	30(23.4)	15(23.8)	15(23.1)	0.92
SMAT	2(1.6)	0(0.0)	2(3.1)	0.15
AMA	5(3.9)	1(1.6)	4(6.1)	0.17
RBB	14(10.9)	8(12.7)	6(9.2)	0.53
R2/RS	14(10.9)	8(12.7)	6(9.2)	0.53
APCA	6(4.7)	2(3.2)	4(6.1)	0.42
LCS	19(14.8)	8(12.7)	11(16.9)	0.50
Granular	16(12.5)	7(11.1)	9(13.8)	0.64
Fine	3(2.3)	1(1.6)	2(3.1)	0.57
GMPA	2(1.6)	0(0.0)	2(3.1)	0.15

**Table 3:** Analysis of specific characteristics of the patients in relation to non-organ specific autoantibody (NOSA) positivity, and in the two groups (CRCG, colorectal cancer group, and OCG, other cancer group).

Variables in	All Patients	CRCG	OCG	*p*
Positive NOSAs	(90/128)	(37/63)	(53/65)	
CIRS Score, M (±SD)	15.4(±4.7)	12.8(±3.8)	17.3(±4.4)	<0.001
CIRS Index, M (±SD)	1.1(±0.3)	0.9(±0.3)	1.2(±0.3)	<0.001
Previous CT/RT, n (%)	28(31.1)	10(27.0)	18(40.0)	<0.64
Male, n (%)	58(64.4)	22(59.4)	36(67.9)	0.55
Stage I, n (%)	11(12.2)	7(18.9)	4(7.5)	0.20
Stage II, n (%)	14(15.6)	4(10.8)	10(18.9)	0.46
Stage III, n (%)	21(23.3)	16(43.3)	5(9.5)	<0.001
Stage IV, n (%)	40(44.4)	10(27.0)	30(56.6)	0.01
Missing Stage	4(4.4)		4(100.0)	
Deceased	66(73.3)	21(56.7)	45(84.9)	0.006
Early stage, n (%) [I + II]	25(27.8)	11(29.7)	14(26.4)	0.91
Advanced stage, n (%) [III + IV]	61(67.8)	26(70.3)	35(66.0)	0.85
Active disease, n (%)	67(74.4)	27(73.0)	40(75.5)	0.98

**Table 4:** The Cox-regression proportional hazards stepwise model estimates show that the hazard rate can be predicted from a linear combination of the covariates CIRS score, stage I-II-III (against stage IV), and a negative autoantibody test on HEp-2 cells.

Covariate	Coefficient	StdErr	Wald χ^2^	HR	95% CI	*p*
CIRS score	0.113	0.0251	20.279	1.120	1.066–1.176	<0.001
STAGE I-II-III	−1.631	0.364	18.876	0.217	0.110–0.436	<0.001
Negative HEp-2	−0.927	0.290	10.194	0.396	0.224–0.699	0.001

## Data Availability

The data presented in this study are available on request from the corresponding author. The data are not publicly available due to ethical restrictions.
